# The Southwestern fringe of Europe as an important reservoir of caprine biodiversity

**DOI:** 10.1186/s12711-015-0167-8

**Published:** 2015-11-05

**Authors:** Amparo M. Martínez, Luis T. Gama, Juan V. Delgado, Javier Cañón, Marcel Amills, Carolina Bruno de Sousa, Catarina Ginja, Pilar Zaragoza, Arianna Manunza, Vincenzo Landi, Natalia Sevane, The BioGoat Consortium

**Affiliations:** Departamento de Genética, Universidad de Córdoba, Córdoba, Spain; CIISA, Faculdade de Medicina Veterinária, Universidade de Lisboa, Lisbon, Portugal; Departamento de Producción Animal, Universidad Complutense de Madrid, Madrid, Spain; Department of Animal Genetics, Center for Research in Agricultural Genomics (CSIC-IRTA-UABUB), Universitat Autònoma de Barcelona, Bellaterra, Barcelona, Spain; Centro de Ciências do Mar, Universidade do Algarve, Instituto de Higiene e Medicina Tropical (UPMM), UNL, Lisbon, Portugal; CIBIO-InBIO, Centro de Investigação em Biodiversidade e Recursos Genéticos, Universidade do Porto, Campus Agrário de Vairão, Vairão, Portugal; Laboratorio de Genética Bioquímica, Facultad de Veterinaria, Universidad de Zaragoza, Zaragoza, Spain

## Abstract

**Background:**

Portugal and Spain, with six and 22 officially recognized caprine breeds, encompass 25 % of the European Union goat census. Many of these populations have suffered strong demographic declines because of competition with exotic breeds and the phasing-out of low income rural activities. In this study, we have investigated the consequences of these and other demographic processes on the genetic diversity, population structure and inbreeding levels of Iberian and Atlantic goats.

**Methods:**

A sample of 975 individuals representing 25 officially recognized breeds from Portugal and Spain, two small populations not officially recognized (Formentera and Ajuí goats) and two ecotypes of the Tinerfeña and Blanca Celtibérica breeds were genotyped with a panel of 20 microsatellite markers. A wide array of population genetics methods was applied to make inferences about the genetic relationships and demography of these caprine populations.

**Results:**

Genetic differentiation among Portuguese and Spanish breeds was weak but significant (F_ST_ = 0.07; *P* < 0.001), which is probably the consequence of their short splitting times and extensive gene flow due to transhumance. In contrast, Canarian goats were strongly differentiated because of prolonged geographic isolation. Most populations displayed considerable levels of diversity (mean H_e_ = 0.65).

**Conclusions:**

High diversity levels and weak population structures are distinctive features of Portuguese and Spanish breeds. In general, these local breeds have a reduced census, but are still important reservoirs of genetic diversity. These findings reinforce the need for the implementation of management and breeding programs based on genetic data in order to minimize inbreeding, maintain overall genetic and allelic diversities and breed identities, while at the same time taking into account the within-breed genetic structure.

**Electronic supplementary material:**

The online version of this article (doi:10.1186/s12711-015-0167-8) contains supplementary material, which is available to authorized users.

## Background

Goat production is a major economic activity in the Mediterranean basin, with key cultural and environmental implications, especially for smallholders and producers in marginal regions. Many local breeds that are well adapted to harsh conditions have been developed throughout the centuries in order to produce milk, meat, leather and fiber products. However, intensification of production systems since the second part of the 20th century has resulted in the demographic regression of many local populations and the concurrent expansion of a few high-producing exotic breeds [1]. Intensive selection programs and artificial insemination have also contributed to erode the genetic reservoir represented by local breeds. Moreover, enhanced transport and communication systems have generated more uniform production environments in which a few transboundary breeds are clearly predominant. These developments have led to growing concerns about the uncontrolled loss of local goat breeds and the erosion of genetic resources that unavoidably result from census decline and increased inbreeding [1]. In Europe, about 7 % of caprine breeds have already disappeared and many more are at the verge of extinction [2]. In the end, the disappearance of these breeds may result in the loss of traits that are essential for adaptation to extensive farming, such as resistance to various diseases and ability to graze on poor pastures [2].

The importance of goat production in the Iberian Peninsula is well illustrated by the fact that it holds nearly 25 % of the caprine census of the European Union [3]. There are six and 22 caprine breeds officially recognized in Portugal and Spain, respectively, which are raised mostly on marginal and forest lands under extensive conditions. Although a few genetic diversity studies have been carried out for some of these breeds [4–7], no comprehensive analysis has been published regarding their overall diversity and genetic structure. In the current study, we aimed at investigating the amount of diversity, population structure, level of inbreeding and genetic relationships across a broad array of local breeds from the Iberian Peninsula and Atlantic and Balearic archipelagos.

## Methods

### Goat sampling

**Fig. 1:**
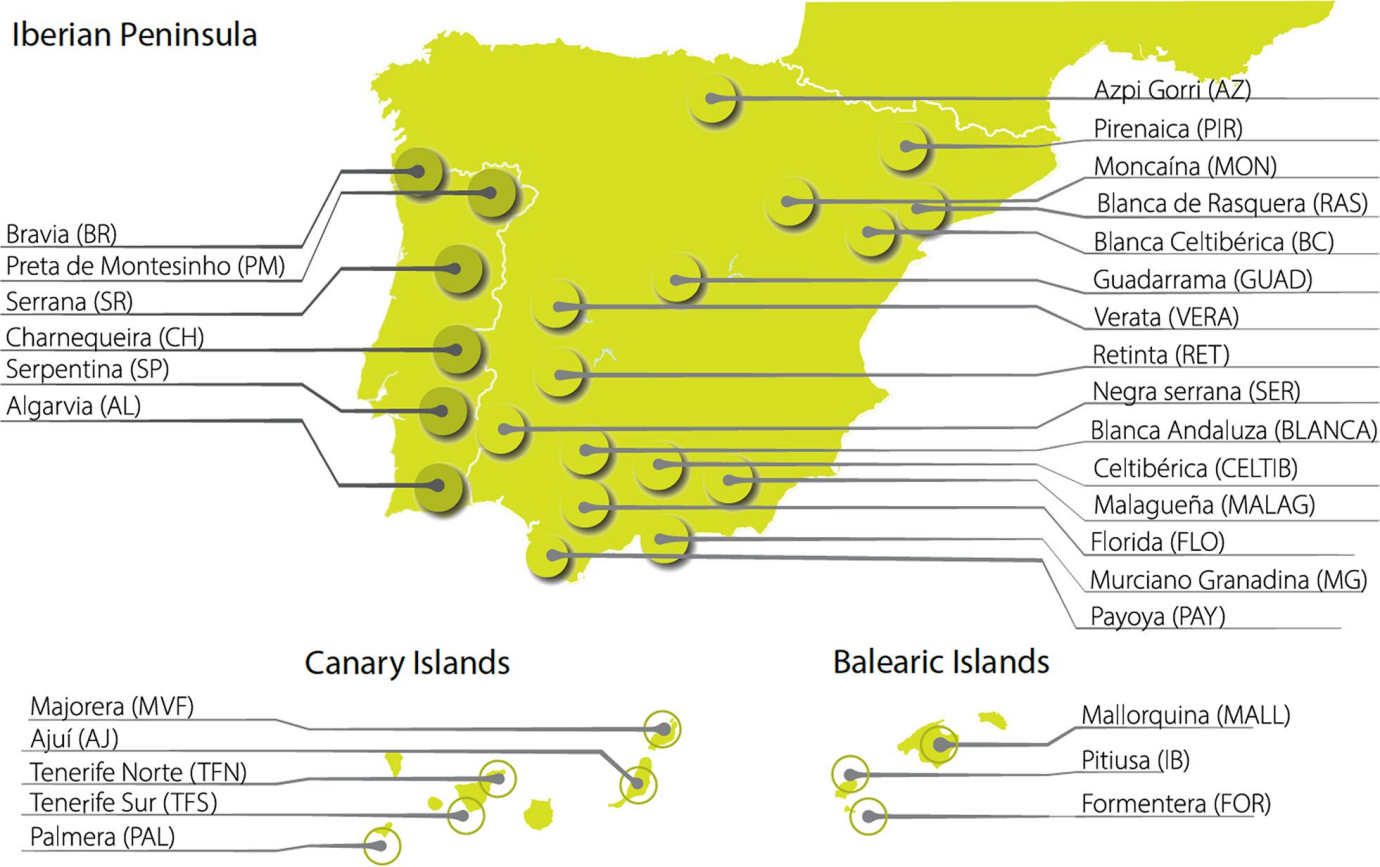
Geographic distribution of 29 goat populations from Portugal and Spain

A total of 975 individuals from 29 local populations in Portugal and Spain were sampled for this study (Table 1; Fig. 1). On average, each breed was represented by about 34 individuals (Table 1), although in the case of the highly endangered Formentera population and Guadarrama breed only 11 animals per population were analyzed. Samples were collected on unrelated individuals registered in Herdbooks (whenever available) from three to 39 herds per breed that covered a wide geographical area. Biological samples (blood or hair roots) were collected by qualified veterinarians during their routine practice, in the framework of official programs that were aimed at identifying, controlling the health and confirming parentage of the populations included in the current work. We studied 25 officially recognized breeds from Portugal and Spain and two small isolated populations that are not yet officially recognized (Formentera and Ajuí goats). Among the breeds sampled, Blanca Celtibérica and Celtibérica are two well-differentiated varieties of the same breed that are reared in very distant areas of the Iberian Peninsula (Fig. 1). Furthermore, we included two Northern and Southern ecotypes of the Tinerfeña breed, which differ in their adaptation to climate i.e., the Southern variety is well adapted to the dry climate typical of South Tenerife Island and the Northern one is raised in the humid and rainy areas of the Northern region of this island [6]. The Murciano-Granadina is officially recognized by the Spanish government as a single breed, although two well differentiated subtypes (Murciano and Granadino) can be distinguished [8]. Finally, it should be mentioned that Formentera and Ajuí goats are not officially recognized in Spain as distinct breeds, but they were included in the study because they are considered as populations with a unique identity in their regions of origin (Balearic and Canary Islands, respectively).

**Table 1:** Genetic diversity parameters estimated with 20 microsatellite loci in 29 Portuguese and Spanish goat populations

Population	Acronym	N/Herds	Census	MNA	Ar^f^	H_e_ ± SD	H_o_ ± SD	F_IS_	NA	HWEd
Spain										
Pirenaica^a^	PIR	18/4	1627^d^	6.60 ± 2.50	5.49	0.695 ± 0.043	0.654 ± 0.027	0.061	0.041	0
Moncaína^a^	MON	32/5	2693^d^	7.10 ± 3.09	5.34	0.687 ± 0.048	0.626 ± 0.020	0.091*	0.048	2
Azpi Gorri^a^	AZ	40/13	1607^d^	6.70 ± 2.81	4.71	0.659 ± 0.039	0.634 ± 0.017	0.039	0.024	0
Blanca de Rasquera^a^	RAS	40/5	5000^d^	6.25 ± 2.81	4.57	0.634 ± 0.050	0.586 ± 0.017	0.077*	0.040	1
Guadarrama	GUAD	11/3	9212^d^	4.50 ± 2.21	–	0.611 ± 0.062	0.551 ± 0.038	0.105	0.039	0
Retinta	RET	15/3	2307^d^	5.61 ± 2.40	–	0.688 ± 0.042	0.677 ± 0.029	0.017	0.026	0
Verata	VERA	28/5	8738^d^	6.50 ± 2.54	4.78	0.654 ± 0.045	0.537 ± 0.022	0.182*	0.077	3
Blanca Andaluza	BLANCA	40/6	8642^d^	6.65 ± 2.62	4.93	0.665 ± 0.041	0.631 ± 0.017	0.052	0.031	0
Celtibérica	CELTIB	40/6	7904^d^	7.15 ± 2.66	5.01	0.663 ± 0.042	0.621 ± 0.017	0.064	0.030	0
Blanca Celtibérica	BC	30/4	<100	6.55 ± 2.26	4.90	0.652 ± 0.044	0.574 ± 0.021	0.123*	0.064	0
Malagueña	MALAG	40/15	40,872^d^	6.80 ± 2.88	5.06	0.683 ± 0.041	0.627 ± 0.017	0.083*	0.042	0
Murciano-Granadina	MG	40/15	99,335^d^	6.6 0 ± 2.37	4.96	0.655 ± 0.049	0.615 ± 0.017	0.062*	0.031	0
Florida	FLO	40/19	24,249^d^	7.25 ± 3.01	5.22	0.697 ± 0.036	0.666 ± 0.017	0.045	0.024	0
Payoya	PAY	36/5	6905^d^	6.40 ± 3.14	4.89	0.672 ± 0.041	0.674 ± 0.017	−0.003	0.024	0
Negra Serrana	SER	40/5	4715^d^	6.35 ± 2.62	4.62	0.657 ± 0.037	0.605 ± 0.017	0.080*	0.045	3
Formentera^b^	FOR	11/3	225^d,g^	4.20 ± 1.61	4.04	0.598 ± 0.051	0.558 ± 0.034	0.070	0.035	0
Pitiusa^b^	IB	40/10		6.35 ± 2.39	4.71	0.652 ± 0.045	0.573 ± 0.017	0.124*	0.061	1
Mallorquina^b^	MALL	40/10	236^d^	6.80 ± 2.61	4.77	0.649 ± 0.040	0.600 ± 0.017	0.077*	0.047	1
Ajuí^c^	AJ	40/-	1700^d^	5.85 ± 2.28	4.44	0.651 ± 0.029	0.618 ± 0.017	0.052	0.034	1
Majorera^c^	MFV	40/10	12,832^d^	6.60 ± 2.89	4.63	0.637 ± 0.038	0.612 ± 0.017	0.039	0.027	0
Palmera^c^	PAL	40/5	9158^d^	4.15 ± 1.53	3.24	0.497 ± 0.038	0.507 ± 0.018	−0.020	0.020	0
Tenerife Norte^c^	TFN	40/5	4705^d,g^	5.30 ± 2.47	4.04	0.603 ± 0.037	0.588 ± 0.018	0.026	0.031	0
Tenerife Sur^c^	TFS	40/8		6.00 ± 2.58	4.16	0.595 ± 0.037	0.590 ± 0.017	0.009	0.012	0
Portugal										
Bravia	BR	39/39	9768^e^	5.95 ± 2.54	4.31	0.632 ± 0.046	0.621 ± 0.017	0.017	0.018	0
Serpentina	SP	30/17	4816^e^	6.80 ± 3.21	4.94	0.671 ± 0.045	0.618 ± 0.020	0.080*	0.043	0
Algarvia	AL	30/29	3991^e^	6.35 ± 2.62	4.75	0.681 ± 0.036	0.647 ± 0.020	0.052	0.036	0
Charnequeira	CH	29/10	4403^e^	6.40 ± 2.33	4.94	0.685 ± 0.034	0.655 ± 0.020	0.044	0.038	0
Serrana	SR	29/26	18,607^e^	6.90 ± 2.83	5.00	0.674 ± 0.042	0.601 ± 0.020	0.110*	0.049	1
Preta de Montesinho	PM	37/13	707^e^	6.75 ± 2.79	4.84	0.669 ± 0.043	0.563 ± 0.019	0.160*	0.068	1
Average		34		6.24	4.71	0.65	0.61	0.039		

*N/Herds* sample size and herds, *MNA* mean number of alleles, *Ar* allelic richness, *H*_*e*_ expected heterozygosity, *H*_*o*_ observed heterozygosity, *SD* standard deviation, *F*_*IS*_ within-breed inbreeding coefficient and significance (* *P* < 0.001), *NA* frequency of null alleles, *HWEd* number of loci not complying with Hardy–Weinberg equilibrium within-breed (*P* < 0.001)

^a^Breeds from the Pyrenean area

^b^Breeds from the Balearic Islands

^c^Breeds from the Canary Islands

^d^ https://aplicaciones.magrama.es/arca-webapp/flujos.html?_flowId=catalogoRazas-flow&_flowExecutionKey=e1s (Census at 12/31/2014 considering registered animals)

^e^ http://www.dgv.min-agricultura.pt/xeov21/attachfileu.jsp?look_parentBoui=3820310&att_display=n&att_download=y (Census at 12/31/2014 considering registered animals)

^f^Allelic richness per locus and population was based on a minimum sample size of nine diploid individuals. The amount of data was not sufficient to calculate allelic richness for Guadarrama and Retinta breeds

^g^Both populations are registered in the same herdbook

### Microsatellite genotyping

Genomic DNA was obtained from hair or blood samples using the Chelex 100 chelating resin (Bio Rad Laboratories, Hercules, CA, USA) according to the methodology described by Walsh et al. [9]. Twenty microsatellite loci were chosen according to the recommendations of the FAO/ISAG [10]. More specifically, the following loci were analyzed: *BM1329, BM6506, BM6526, BM8125, CRSM60, CSRD247, ETH010, ETH225, HAUT27, ILSTS011, INRA063, MAF065, MAF209, McM527, MM12, OarFCB048, OarFCB304, SPS115, SRCRSP08,* and *TGLA122* (see Additional file 1: Table S1). Each multiplex PCR was carried out in 25 µL reaction tubes containing 30–60 ng of genomic DNA, 5 µL of 2× PCR mastermix and 2 pmol of each primer (forward primer labeled at the 5′ end with HEX, FAM or NED fluorescent dyes). The PCR mix was subjected to an initial denaturation step at 95 °C for 5 min, followed by 35 cycles of 45 s at 95 °C, 35 s at 55 °C and 35 s at 72 °C with a final extension step of 10 min at 72 °C. PCR products were separated by electrophoresis using an ABI377 equipment (Applied Biosystems, Madrid, Spain) according to the manufacturer’s recommendations. Allele sizes were determined with the internal size standard GeneScan-400HD ROX (Applied Biosystems, Madrid, Spain). Reference samples were used in each assay to ensure the consistency of allele assignments.

### Population genetics analyses

Allele frequencies for each locus, total number of alleles per locus (NA), observed (H_o_) and unbiased expected (H_e_) heterozygosities, and mean number of alleles (MNA) per population were calculated using the MICROSATELLITE TOOLKIT software [11]. Weir and Cockerham [12] F-statistics and allelic richness across all loci per breed were estimated with FSTAT [13]. A graphic representation of the matrix containing pairwise *F*_ST_ distances derived from the 29 goat populations studied was generated with R-lequin [14]. The mean *F*_ST_ and their 95 % confidence interval across loci after 1000 bootstraps were calculated using the GENETIX 4.05 software [15]. The same program was used to calculate *F*_ST_*P* values after 1000 permutations of individuals across the entire population. A Mantel test [16] was performed using the statistical software R version 2.15.0 [17] to test for isolation-by-distance, by investigating the correlation that exists between genetic and geographical distances between pairs of breeds. Fisher’s exact tests for ascertaining Hardy–Weinberg (HW) equilibrium across loci and populations and estimation of the frequencies of null alleles were performed with GENEPOP 1.2 [18]. The relationship between the percentage of missing data for each locus and *F*_IS_ was examined [19] with the Spearman’s rank correlation using the SAS statistical package v. 9.1.3 [20]. A positive relationship between *F*_IS_ and missing data for a given locus indicates that amplification failure is due to individuals that carry a null allele in the homozygous state.

The model-based clustering program STRUCTURE [21] was used to investigate population structure and to estimate the proportions of individual genotypes derived from the inferred clusters. We considered an admixture model and uncorrelated allele frequencies. The putative number of clusters (K) ranged from 2 to 35, and five independent runs were performed with a number of Monte-Carlo Markov chain iterations that ranged from 250,000 (50,000 burn-in length) for K between 2 and 10, to 500,000 (150,000 burn-in length) for K between 11 and 35. To identify the most probable number of ancestral populations, we investigated the mean and variance of the likelihood plots of the data for different K-values (i.e., plot of ln Pr(X/K) vs. K). Bar charts representing the proportions of the genotype membership coefficient of each individual (*q*) in a given ancestral population, as obtained with STRUCTURE, were visualized using DISTRUCT [22]. The degree of admixture or ancestry diversity of each breed was calculated as 1 − Σ(q_k_)^2^, where *q*_*k*_ is the average fraction of the genetic ancestry of a given breed that belongs to the *k*th ancestral population, estimated by STRUCTURE analyses [23]. The correlation between the proportion of mixed ancestry and the expected heterozygosity was calculated to evaluate the relationship between admixture and within-breed diversity. Average genotype membership coefficients in each cluster (*Q*) were converted into genetic distances among breeds, following the methodology described by Cañón et al. [24]. In this case, a FORTRAN program that implements the computation of Weitzman’s diversity was used, and with the ultra-metric distance matrix obtained, a hierarchical tree resulting from Weitzman’s algorithm [25] was constructed with MEGA 5 [26]. Information on genetic markers was combined with spatial data to draw synthetic contour maps of the Iberian Peninsula, representing the geographical patterns of genetic variability [27, 28]. These maps were drawn based on the interpolation of genetic contributions to each breed, as computed in the analysis with STRUCTURE for K = 3. The kriging interpolation method was used [29] and the graphical library of statistical software R [17] was used to display the maps. Each breed was represented on the map by the coordinates of its center of geographical dispersion.

Genetic structure was further investigated by a factorial analysis of correspondence (FAC) using the function “AFC 3D by populations” implemented in GENETIX 4.05 [15], which is analogous to a principal component analysis. In this approach, allele frequencies are used to infer the relative position of each breed based on Chi square distances.

## Results

### Genetic diversity

The 20 microsatellite markers analyzed were highly variable, with a total of 236 alleles across all populations. The number of alleles per locus ranged from 3 (*MAF209*) to 22 (*OarFCB304*), with an average of 11.8 (see Additional file 2: Table S2). Expected (H_e_) and observed (H_o_) heterozygosities per locus across all breeds ranged from 0.199 (*ETH225*) to 0.86 (*MM12*), and from 0.20 (*ETH225*) to 0.80 (*MM12*), respectively. The overall means across loci and breeds for H_e_ and H_o_ were equal to 0.65 and 0.61, respectively (see Additional file 2: Table S2). Among the breeds studied, the smallest MNA per locus (<5.0) was found for the Palmera, Formentera and Guadarrama goat breeds, and the largest MNA (>7.0) for Celtibérica, Florida and Moncaína (Table 1). Palmera had the lowest mean allelic richness (3.2), while the highest estimate was found for Pirenaica (5.5) and Moncaína (5.3). The amount of data was not sufficient to calculate allelic richness for the Guadarrama and Retinta breeds because some individuals were not successfully amplified and genotyped at all microsatellite markers and thus, there were many blanks in the dataset. The H_e_ and H_o_ by breed, averaged across loci, and the measure of within-breed discrepancy among loci (*F*_IS_) are in Table 1. H_e_ per breed ranged from 0.50 (Palmera) to 0.70 (Florida and Pirenaica). Of the 29 populations studied, 12 exhibited a highly significant deficiency in heterozygotes (*P* < 0.001) and, in this regard, the Verata (0.18) and Preta de Montesinho (0.16) breeds showed the highest within-breed *F*_IS_ coefficients (Table 1). Only two breeds (Verata and Negra Serrana) had more than two loci that deviated from HW equilibrium (Table 1), and only two microsatellite markers (*OarFCB304* and *SPS115*) did not adjust to the HW equilibrium model for more than one breed (*P* < 0.001), as shown in Additional file 2: Table S2.

Frequencies of null alleles ranged from 1.2 % for Tenerife Sur to 7.7 % for Verata breeds (Table 1). In a locus-by-locus analysis, the markers with the highest frequencies of null alleles were *SPS115* (9.7 %) and *INRA063* (6.6 %) (see Additional file 2: Table S2). Moreover, the relationship between the percentage of missing data for each locus and *F*_IS_ was not significant (*P* > 0.01).

### Measurement of Wright’s *F*_ST_ coefficient

**Fig. 2:**
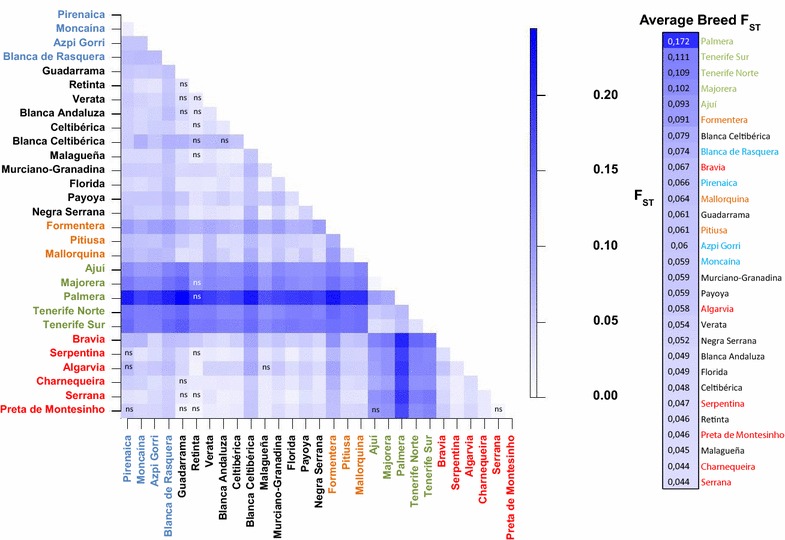
Graphical representation of pairwise *F*_ST_ distances between the 29 goat populations studied. *Color-codes* are defined on the scale at the *right side* of the figure. *ns* not significant, *blank* significant (*P* < 0.001)

The *F*_ST_ averaged across loci (see Additional file 2: Table S2) resulted in mean *F*_IS_ estimates across populations of 0.06 (CI 0.05 to 0.08, data not shown), while the amount of differentiation among populations (*F*_ST_ = 0.07, CI 0.06 to 0.09, data not shown) was significant (*P* < 0.001), and the inbreeding coefficient of individuals relative to the total population (*F*_IT_) was equal to 0.13 (CI 0.12 to 0.15, data not shown). The assessment of genetic distances among breeds based on *F*_ST_ values (Fig. 2) revealed a weak level of differentiation among Portuguese and Spanish goats. In contrast, a strong genetic differentiation was observed between Canarian and Iberian goats.

The Mantel test showed a strong correlation (R^2^ = 0.72) between *F*_ST_ genetic distances and geographic distances among breed pairs (*P* < 0.0001 after 10,000 permutations), although this value dropped to 0.029 when Canarian breeds were removed from the analysis (see Additional file 3: Figure S1). Thus, for breeds located in the Iberian Peninsula there was no clear association between genetic and geographical distances.

### Structure analysis and tree building

Microsatellite data for the goat populations studied were analyzed with the Bayesian model-based clustering implemented in STRUCTURE [21] and the plot of average likelihoods of the data for different values of K (ln Pr(X/K) vs. K) was used to infer the most likely number of genetic clusters, which was determined to be 12 (see Additional file 4: Figure S2). The proportional contributions of the inferred ancestral populations per breed are in Additional file 5: Table S3 for K = 12.

**Fig. 3:**
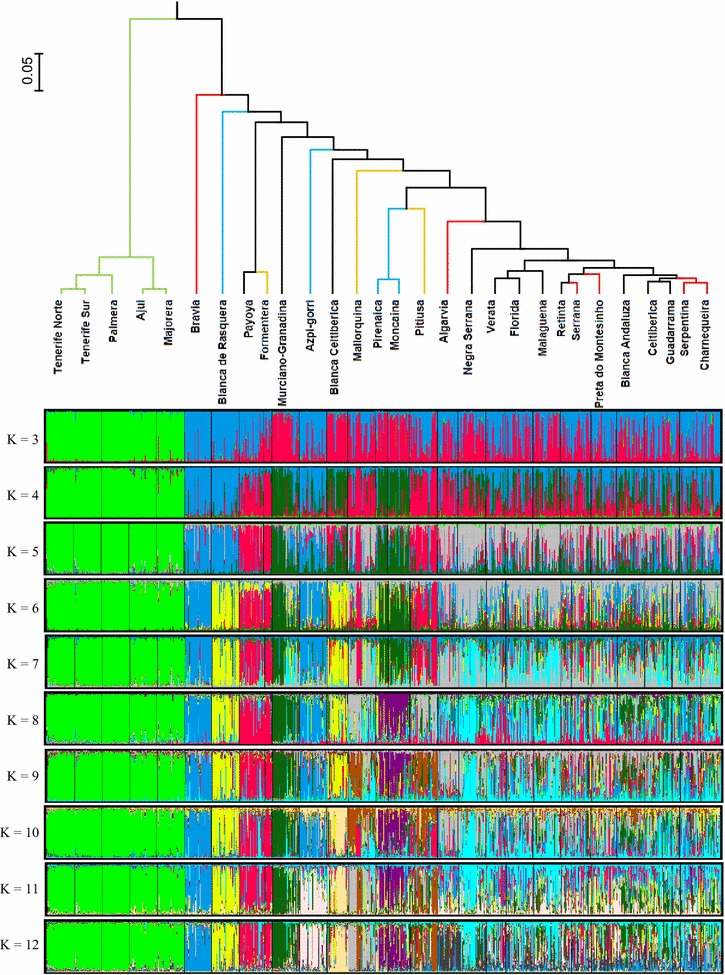
Clustering of 29 goat populations from Portugal and Spain with STRUCTURE. *Top* UPGMA clustering of the ultra-metric distance matrix obtained from the conversion of the average genotype membership coefficients (*Q*) in each cluster into genetic distances for K = 12. *Bottom* Graphic representation of the estimated individual membership coefficients (*q*) as inferred with STRUCTURE, assuming a number of ancestral populations K ranging from 3 to 12

The estimated individual genotype membership coefficients in each ancestral population for a number of genetic clusters K ranging from 3 to 12 are graphically represented in Fig. 3. In particular, the five Canarian breeds were clearly assigned to a single cluster, which was maintained even at K = 24 (see Additional file 6: Table S4). This result indicates that the Canarian populations are strongly differentiated from those of the Iberian Peninsula and the Balearic Islands.

To gain additional insights into population structure, the fractional contributions of ancestral populations to each breed were converted into genetic distances among breeds and represented in UPGMA trees for K = 3 (see Additional file 7: Figure S3) and K = 12 (Fig. 3), as described by Cañón et al. [24] and García et al. [25]. These analyses showed three main groups for K = 3: (1) Canarian breeds; (2) Blanca de Rasquera, Payoya, Pirenaica, Formentera, Moncaína, and Blanca Celtibérica; and (3) a main group including the remaining breeds (see Additional file 7: Figure S3).

**Fig. 4:**
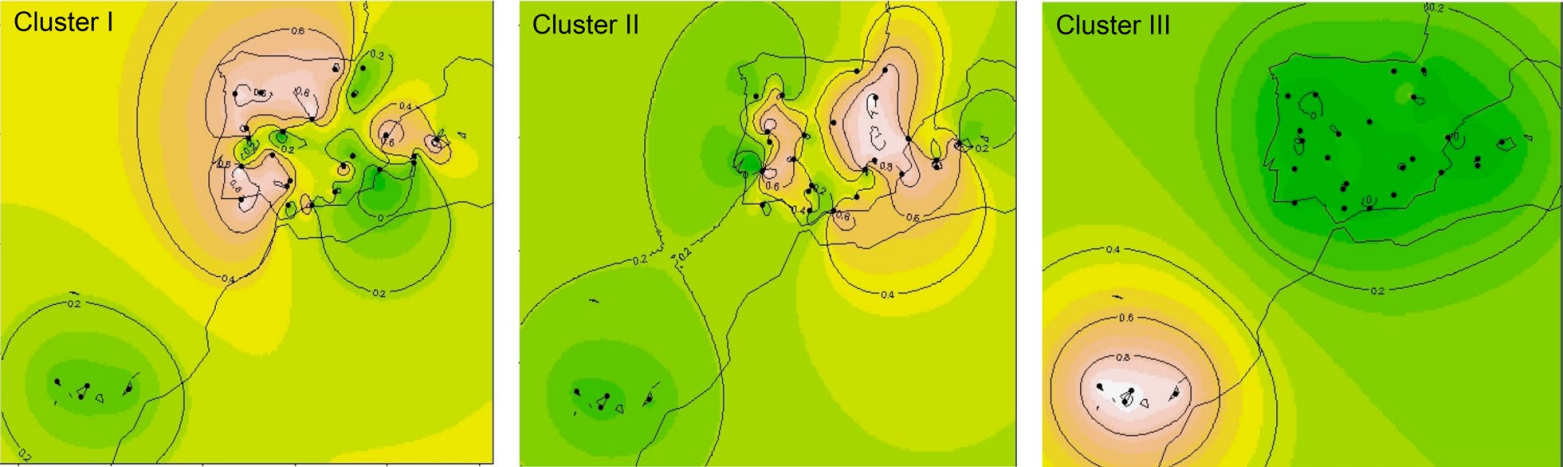
Two-dimension maps obtained by spatial interpolation of the ancestral contribution coefficients (*Q* values) for genetic clusters I, II and III obtained with STRUCTURE for K = 3. Each sampled breed is represented by a *black dot* placed at the center of its geographical dispersion (see Fig. 1 for breed names). *Colors* in the maps indicate the degree of genetic similarity among the breeds sampled, such that breeds sharing a *lighter color* (decreasing from *white* to *pink* and then to *orange*) have a higher contribution of the indicated cluster, while breeds with a *darker color* (*dark green*) do not share a contribution from that cluster

The geographical representation of the interpolation of admixture coefficients (*Q* matrix) for K = 3 (Fig. 4) also indicated the strong differentiation of the Canary group (cluster III), and suggested two main geographical patterns of genetic dispersion in the Iberian Peninsula, one that encompasses the Atlantic breeds, mostly from the northwest and southwest of the Peninsula (cluster I), and another that spreads through the center and east, including both continental and Mediterranean breeds (cluster II).

The tree resulting from the ultra-metric distance matrix obtained with the Weitzman’s algorithm for K = 12 (Fig. 3) indicates that only four groups can be clearly identified, i.e.: (1) Canary Islands breeds; (2) a large group including the Spanish breeds Verata, Florida, Malagueña, Retinta, Blanca Andaluza, Celtibérica, and Guadarrama, and the Portuguese breeds Serrana, Preta de Montesinho, Serpentina, and Charnequeira; (3) the Pirenaica/Moncaína group; and (4) the Payoya/Formentera group. The populations that are in each group (iii) and (iv) shared the same genetic background, with average membership proportions of about 0.5 and 0.7, respectively (see Additional file 5: Table S3). It should be noted that the remaining breeds (Blanca de Rasquera, Murciano-Granadina, Negra Serrana, Pitiusa, Mallorquina and Algarvia) had an estimated membership fraction greater than 0.4 in their main respective inferred clusters (see Additional file 5: Table S3). The clustering of the Azpi Gorri breed was cryptic and shared its main genetic background inconsistently with either Blanca Celtibérica or Bravia goats depending on which STRUCTURE run was considered.

A highly significant positive correlation (r = 0.71, P < 0.0001) was estimated between breed H_e_ and levels of admixture based on global STRUCTURE results for K = 12 (see Additional file 5: Table S3), which suggested that the levels of admixture of breeds could be responsible for a large proportion of their estimated genetic variability.

### Factorial analysis of correspondence

**Fig. 5:**
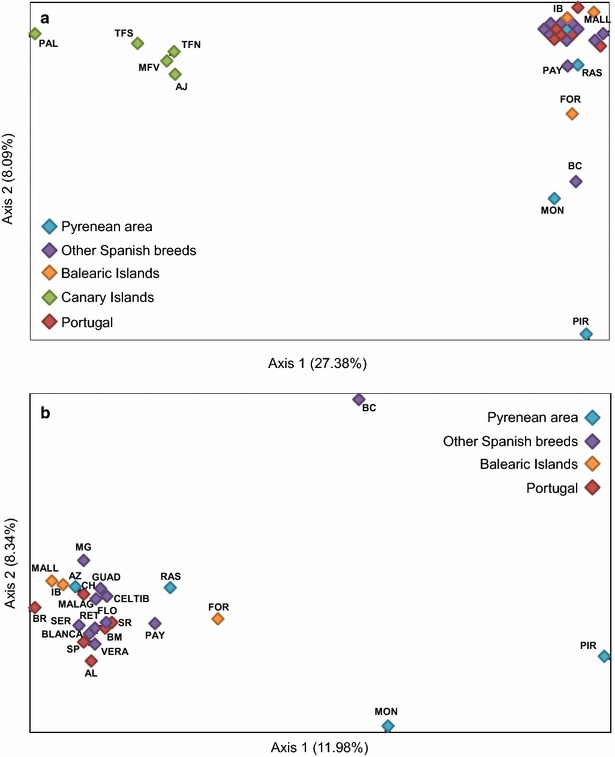
Spatial representation of the tridimensional factorial correspondence analysis (FCA) carried out with GENETIX. **a** 29 goat breeds, **b** Portuguese and Spanish breeds after removing the Canary Islands populations. Breed acronyms: *PIR* Pirenaica, *MON* Moncaína, *AZ* Azpi Gorri, *RAS* Blanca de Rasquera, *GUAD* Guadarrama, *RET* Retinta, *VERA* Verata, *BLANCA* Blanca Andaluza, *CELTIB* Celtibérica, *BC* Blanca Celtibérica, *MALAG* Malagueña, *MG* Murciano-Granadina, *FLO* Florida, *PAY* Payoya, *SER* Negra Serrana, *FOR* Formentera, *IB* Pitiusa, *MALL* Mallorquina, *AJ* Ajuí, *MFV* Majorera, *PAL* Palmera, *TFN* Tenerife Norte, *TFN* Tenerife Sur (TFN), *BR* Bravia, *SP* Serpentina, *AL* Algarvia, *CH* Charnequeira, *SR* Serrana, *PM* Preta de Montesinho

The genetic structure of the 29 Portuguese and Spanish breeds was further assessed with FAC clustering methods. When all breeds were included in the analysis, the first two axes contributed 27.4 and 8.1 % of the total inertia, respectively (Fig. 5a). The Canarian populations were again clearly separated from the main group and accounted by themselves for 79.3 % of the total inertia on Axis 1. On Axis 2, the most differentiated breeds were Pirenaica and Moncaína, explaining 33.5 % of the total inertia, followed by Blanca Celtibérica (15.8 %). Canarian goats were excluded in a second analysis that was aimed at investigating with more detail the relationships amongst Peninsular and Balearic breeds (Fig. 5b). In this case, Axes 1 and 2 contributed with 12 and 8.3 % of the total inertia, respectively, and most breeds were clustered in a single cluster, but the major proportion of the variation on both axes was again explained by the Pirenaica, Moncaína and Blanca Celtibérica breeds.

## Discussion

### A weak population structure in goat populations from the Iberian Peninsula and Balearic archipelago

We studied 29 autochthonous goat populations that were sampled in Portugal and Spain by using 20 microsatellite markers in a survey that included endangered populations (e.g., Pitiusa and Blanca Andaluza), commercial goat breeds with larger census (e.g., Murciano-Granadina and Malagueña) and a feral population from the Canary Islands (Ajuí). Estimates of genetic diversity and analyses of population structure with microsatellites were previously reported for certain specific Iberian goat populations [6, 7, 30] but, to our knowledge, this study is the most comprehensive analysis of the genetic diversity of native goats from these two countries. This broad representativeness offers an unprecedented perspective about the variation and population structure of Portuguese and Spanish goats. One of the major features revealed by our study is that the population structure of Iberian goats is weak, and that populations from Portugal and Spain (with the exception of Canarian goats) are, in general, poorly differentiated. This observation can be explained by two factors. First, the splitting times of Portuguese and Spanish breeds are probably very short (in the range of a few hundred years) precluding a strong genetic differentiation. This is well-known for sheep breeds such as the Merino, which was maintained as a joint population exclusively in the Iberian Peninsula until the 18th century [31]. Certain goat populations may have been subject to similar handling procedures. Second, there are no strong geographical barriers between Portugal and Spain that would hinder a bidirectional gene flow. Indeed, transhumance trails crossed the border that separates both countries [32], particularly in the southern part of the Iberian Peninsula, and small ruminants would be periodically taken from one side to the other, providing the opportunity for genetic exchange to occur. Within Portugal and Spain, seasonal pastoralism and transhumance, which were typical of most goat production systems in the Iberian Peninsula until the mid-20th century, contributed to weaken the population structure [33]. It has also been claimed that long-distance cyclic migrations and the great mobility of goats are the main causal factors that explain the poor phylogeographic structure detected with mitochondrial markers in the Iberian Peninsula [5] and at a worldwide scale [34]. In our study, it is interesting to highlight that the genetic affinity between the breeds in Cluster II of Fig. 4 (which reveals the existence of two clouds of affinity in the center and west of the Iberian Peninsula, respectively) follows a clear north to south dispersion path. Since it is known that nearly all the historical transhumance trails also followed the same direction [32], it is expected that the observed geographic relationship among breeds probably reflects this gene flow and admixture with migrants along the routes of transhumance.

In several instances, the between-breed genetic affinities reported in our study were quite unexpected. For example, the Payoya breed (from the southern Mediterranean coast of the Peninsula) clustered with the Formentera population (from the Balearic Islands) in the absence of any historical record that would justify such a relationship. However, an in-depth analysis of this cluster allowed for a clear separation of the two breeds, thus suggesting that migration to the Balearic Islands may have occurred in the past. A close relationship was expected to exist between the Moncaína and Guadarrama breeds, given their very close geographic distribution and common origin, and the fact that these two populations are officially considered as varieties of the Pirenaica breed. However, the analysis with STRUCTURE revealed a close relationship between Pirenaica and Moncaína goats, while the Guadarrama breed was clearly separated from both populations.

We were unable to detect a clear differentiation between several of the breeds analyzed (Figs. 3, 5), an outcome that may have demographic and technical causes. The availability of high-density panels of single nucleotide polymorphisms (SNPs), which until now have been very little used for population genetic studies in goats, will allow a more refined analysis of goat population structure, and contribute to a better understanding of the recent evolutionary history of domestic goats [35–37].

### Canarian goats are strongly differentiated from their Iberian and Balearic counterparts

The Canarian breeds showed a strong genetic differentiation from their Iberian and Balearic counterparts, probably because they descend from the North African goat populations that were introduced by the first Imazighen settlers of the Canary Islands [38, 39]. After the conquest by the Spaniards in the 15th century, the Canary archipelago became an important maritime trading platform between Europe and the Americas [39], providing the opportunity for Canarian goats to hybridize with foreign breeds from Spain and other European and African countries. Notably, the intensity of this hybridization process may have varied from one island to another [40].

### Existence of genetic substructure at the within-breed level

The existence of within-breed genetic heterogeneity should be understood as an essential part of their history, instead of considering it as a negative feature [41]. Several examples of within-breed genetic heterogeneity were identified in our study. The Murciano-Granadina breed is one of the most important dairy goat populations in Spain and results from the administrative unification, in the 1970’s, of two well differentiated genetic groups [8]. This event may explain the substructure revealed by the significant *F*_IS_ estimate obtained for this breed (Table 1). A somewhat similar situation was evidenced in the Serrana breed, which has three distinct ecotypes that are raised in different geographical regions i.e. Transmontana, Serra and Ribatejana. This geographical isolation may have facilitated the emergence of a population substructure resulting in the significant deficiency in heterozygotes observed in our study. Another case is represented by the Formentera goats, which are classified as part of the Pitiüsa breed in the Spanish Official Breed Catalogue [38]. However, our findings indicate that Formentera and Pitiüsa goats are clearly differentiated (Figs. 3, 5), probably because of genetic drift and inbreeding. In other breeds such as Moncaína, Verata, Serpentina and Preta de Montesinho (Table 1), factors such as inbreeding, population substructure and genetic drift resulting from small population sizes could also have contributed to the observed deficiency in heterozygotes, although the possibility of population substructure cannot be excluded. Finally, the Celtibérica and Blanca Celtibérica populations are considered as two geographic varieties of the same breed, but Celtibérica is reared mainly in the Southern Central part of the Iberian Peninsula while Blanca Celtibérica is raised in Eastern Spain (Castellón province), along the Mediterranean coast. Some decades ago, Blanca Celtibérica was considered extinct [38] but a few breeders still maintain this breed in the Castellón region. All our analyses indicated a clear separation between these two populations, and Blanca Celtibérica goats were more differentiated from the remaining Iberian breeds than Celtibérica (Figs. 3, 5), which showed some level of genetic affinity with Andalusian goats (Blanca Andaluza, Malagueña and Florida).

### Portuguese and Spanish goats display high levels of diversity

In the last decades, many caprine local breeds have suffered a strong demographic decline because of the phasing-out of low income farming activities, replacement of local breeds by cosmopolitan high-producing breeds, intensification of agricultural practices, and the broad use of artificial insemination (AI) as well as other factors [2]. AI is only used for a few Spanish breeds such as the Murciano-Granadina, Malagueña, Florida breeds and the endangered Payoya and Pitiusa breeds and it is always applied within the framework of the selection or conservation programs of these breeds (see Additional file 8: Table S5). In Portugal, AI is used on a small scale only in the Serrana breed, but all goat breeds have germplasm conservation programs, including semen cryopreservation (see Additional file 8: Table S5).

Overall, we found that genetic diversity was high within most goat breeds studied here, with an overall mean of ~12 alleles/locus and an average H_e_ of 0.65 for all microsatellite loci analyzed. At the breed level, the mean number of alleles per locus was about 6, with an allelic richness corrected for sample size of about 5 and a H_e_ of about 0.65. These values are greater than those found for Asian goats [42], and similar to those reported for European, Chinese, Indian, and Brazilian breeds [24, 43–46]. However, these comparisons are not straightforward, because they depend on the panel of genetic markers used, the breeds analyzed and sample size (which has a strong effect on the number of alleles detected).

Among the Portuguese and Spanish goat breeds, 12 out of 29 showed significantly positive *F*_IS_ values, which indicates a deficiency in heterozygotes that could be due to inbreeding, the Wahlund effect (population substructure), and other causes [47]. The Verata, Preta de Montesinho, Pitiüsa and Serpentina breeds exhibited the highest *F*_IS_ values, but at present it is difficult to infer if this observation is due to population structure, inbreeding or a combination of both factors. As previously said, in the Pitiüsa population, the census is rather small, so inbreeding may have contributed significantly to the high *F*_IS_ that we detected (Table 1). In contrast, in the Verata breed (*F*_IS_ = 0.183), there are over 8000 breeding females and the rather high *F*_IS_ observed may reflect the existence of population substructure.

Overall, we found that the levels of diversity were high, although this finding should not give rise to an excessive optimism because many of the breeds that we analyzed are undergoing a steady and sustained demographic decline that may lead, in the worst case scenario, to their disappearance. One of the most compelling cases is the Blanca de Rasquera population, which in the first half of the 20th century had a census of 30,000 individuals [48], whereas today it has decreased to 5000 goats. In Portugal and Spain, as in many other Western countries, the proportion of the economic active population dedicated to farming activities is decreasing at a rapid pace, and sheep and goats have been progressively displaced to marginal areas, while local genetic resources are being replaced by more productive industrial and transboundary breeds. Moreover, intensive selection schemes, population fragmentation into discrete subpopulations and artificial insemination contribute to reduce the variability of local as well as transboundary breeds. The joint effects of all these threats could significantly shrink the current gene pool of goat breeds and cause a loss of biodiversity that may have deleterious effects on adaptive traits such as resistance to various diseases, adaptation to harsh environments and the ability to cope with climate changes.

## Conclusions

Our results indicate that the levels of genetic diversity are high in the native goat breeds from Portugal and Spain. Indeed, local breeds, often with a small census, are important reservoirs of genetic diversity. With regard to population structure, Portuguese and Spanish breeds were weakly differentiated. In contrast, Canarian breeds showed a strong genetic differentiation from their Iberian and Balearic counterparts, probably due to influences from their North African counterparts combined with continued geographic isolation. Among the Portuguese and Spanish populations, 12 out of 29 showed significantly positive *F*_IS_ values, which could be due to a variety of reasons, such as inbreeding and the Wahlund effect. Although, overall, the levels of diversity that we observed are high, these results should be taken with caution, because many of the breeds analyzed are undergoing a steady and sustained demographic decline that could lead to their disappearance. Management and breeding programs based on genetic data should be undertaken in order to minimize inbreeding, maintain overall genetic and allelic diversities and breed identities, and also account for within-breed genetic structure.

## Additional files

10.1186/s12711-015-0167-8 Microsatellites analyzed in this study. This file contains data on the microsatellites analyzed i.e. chromosome number, GenBank accession number, primer sequences, fluorescent dye, multiplex and allele range.
10.1186/s12711-015-0167-8 Descriptive statistics of the genetic diversity of the 20 microsatellite markers used to genotype the 29 goat populations. This file contains data on the microsatellites analyzed i.e. total number of alleles (NA); expected heterozygosity (He); observed heterozygosity (Ho); Wright’s F- statistics and number of breeds showing deviations from Hardy–Weinberg equilibrium (HWEd) (P < 0.001).
10.1186/s12711-015-0167-8 Correlation between *F*_ST_ genetic distances and geographic distances (*P* > 0.0001 after 10,000 permutations). (a) 29 goat breeds. (b) Portuguese and Spanish breeds after removing the Canary Islands populations.
10.1186/s12711-015-0167-8 Estimated posterior probabilities of the data for different values of K in the analysis with STRUCTURE. This file contains the plots of the average likelihood of data (ln Pr(X|K)) (mean ± standard deviation of five independent runs) obtained with STRUCTURE for 29 goat breeds from Portugal and Spain for two to 35 inferred genetic clusters (K).
10.1186/s12711-015-0167-8 Proportional contribution of the clusters inferred with STRUCTURE (K = 12) to the gene pools, ancestry diversity and expected heterozygosities of 29 Portuguese and Spanish goat breeds.
10.1186/s12711-015-0167-8 Proportional contribution of the clusters inferred with STRUCTURE (K = 24) to the gene pool of the 29 Portuguese and Spanish goat populations. Contributions of the most important clusters per population are represented in bold. Genetic clusters with a contribution of less than 10 % in any of the 29 goat populations are shown in grey.
10.1186/s12711-015-0167-8 UPGMA clustering of the ultra-metric distance matrix obtained from the conversion of the average genotype membership coefficients (Q) in each cluster (K = 3) into genetic distances.
10.1186/s12711-015-0167-8 Demographic information on the 29 Portuguese and Spanish goat populations. This file includes the name of the populations, acronyms used, existence of Herdbook, year of the establishment of the Herdbook, census, geographic distribution (GD), use of artificial insemination (AI), existence of gene bank (GB), existence of conservation programs (CP) and existence of selection programs (SP). Source: http://www.magrama.gob.es/es/ganaderia/temas/zootecnia/razas-ganaderas/razas/catalogo/ (Accessed on 15 Aug 2015).
