# Supplementary material for: The Southwestern fringe of Europe as an important reservoir of caprine biodiversity
Source: Genet Sel Evol. 2015 Nov 5;47:86. doi: 10.1186/s12711-015-0167-8 (PMC4635977; doi:10.1186/s12711-015-0167-8)
Supplement: Supplementary file 1 — 10.1186/s12711-015-0167-8 Microsatellites analyzed in this study. This file contains data on the microsatellites analyzed i.e. chromosome number, GenBank accession number, primer sequences, fluorescent dye, multiplex and allele range. [file 12711_2015_167_MOESM1_ESM.pdf]

Additional file 1 Table S1. Microsatellites analyzed in this study.

Marker	Chromosome	Genbank (Accession Number)	Primer Sequences (5' - 3')	Fluorochrome	Multiplex	Allele range (bp)
BM6506	OAR1 (Ovis aries)	-	GCA CGT GGT AAA GAG ATG GC<br>AGC AAC TTG AGC ATG GCA C	HEX	I	185-230
CSRD247	OAR14 (Ovis aries)	-	GGA CTT GCC AGA ACT CTG CAA T<br>CAC TGT GGT TTG TAT TAG TCA GG	FAM	I	220-247
ETH225	BTA9 (Bos taurus)	Z14043	GAT CAC CTT GCC ACT ATT TCC T<br>ACA TGA CAG CCA GCT GCT ACT	NED	I	131-159
INRA063	CHI18 (Capra hircus)	X71507	ATT TGC ACA AGC TAA ATC TAA CC<br>AAA CCA CAG AAA TGC TTG GAA G	HEX	I	164-186
TGLA122	BTA21 (Bos taurus)	-	CCC TCC TCC AGG TAA ATC AGC<br>AAT CAC ATG GCA AAT AAG TAC ATA C	FAM	I	220-247
BM8125	OAR17 (Ovis aries)	G18475	CTC TAT CTG TGG AAA AGG TGG G<br>GGG GGT TAG ACT TCA ACA TAC G	FAM	II	110-130
HAUT27	BTA26 (Bos taurus)	X89252	TTT TAT GTT CAT TTT TTG ACT GG<br>AAC TGC TGA AAT CTC CAT CTT A	NED	II	120-158
ILSTS011	BTA14 (Bos taurus)	L23485	GCT TGC TAC ATG GAA AGT GC<br>CTA AAT TGC AGA GCC CTA CC	HEX	III	256-294
SPS115	BTA15 (Bos taurus)	FJ828564	AAA GTG ACA CAA CAG CTT CTC CAG<br>AAC GAG TGT CCT AGT TTG GCT GTG	NED	III	234-258
BM6526	BTA26 (Bos taurus)	-	CAT GCC AAA CAA TAT CCA GC<br>TGA AGG TAG AGA GCA AGC AGC	FAM	IV	150-190
MM12	BTA9 (Bos taurus)	Z30343	CAA GAC AGG TGT TTC AAT CT<br>ATC GAC TCT GGG GAT GAT GT	NED	V	101-145

BM1329	OAR6 (Ovis aries)	G18422	TTG TTT AGG CAA GTC CAA AGT C<br>AAC ACC GCA GCT TCA TCC	NED	VI	160-182
MAF065	OAR15 (Ovis aries)	M67437	AAA GGC CAG AGT ATG CAA TTA GGA G<br>CCA CTC CTC CTG AGA ATA TAA CAT G	NED	VI	116-158
MAF209	CHI17 (Capra hircus)	M80358	GAT CAC AAA AAG TTG GAT ACA ACC GTG G<br>TCA TGC ACT TAA GTA TGT AGG ATG CTG	HEX	VI	100-104
OarFCB304	OAR19 (Ovis aries)	L01535	CCC TAG GAG CTT TCA ATA AAG AAT CGG<br>CGC TGC TGT CAA CTG GGT CAG GG	HEX	VI	150-188
McM527	OAR5 (Ovis aries)	L34277	GTC CAT TGC CTC AAA TCA ATT C<br>AAA CCA CTT GAC TAC TCC CCA A	NED	VII	165-187
SRCRSP8	unknown	L22200	TGC GGT CTG GTT CTG ATT TCA C<br>CCT GCA TGA GAA AGT CGA TGC TTA G	NED	VII	215-255
CSRM60	BTA10 (Bos taurus)	Z30343	AAG ATG TGA TCC AAG AGA GAG GCA<br>AGG ACC AGA TCG TGA AAG GCA TAG	NED	VIII	79-115
ETH010	CHI5 (Capra hircus)	Z22739	GTT CAG GAC TGG CCC TGC TAA CA<br>CCT CCA GCC CAC TTT CTC TTC TC	FAM	IX	200-210
OarFCB048	CHI17 (Capra hircus)	M82875	GAG TTA GTA CAA GGA TGA CAA GAG GCA C<br>GAC TCT AGA GGA TCG CAA AGA ACC AG	FAM	IX	149-173

Chromosome, GeneBank accession number, primer sequences, fluorescent dye, multiplex and allele range.
