# Supplementary material for: The Southwestern fringe of Europe as an important reservoir of caprine biodiversity
Source: Genet Sel Evol. 2015 Nov 5;47:86. doi: 10.1186/s12711-015-0167-8 (PMC4635977; doi:10.1186/s12711-015-0167-8)
Supplement: Supplementary file 2 — 10.1186/s12711-015-0167-8 Descriptive statistics of the genetic diversity of the 20 microsatellite markers used to genotype the 29 goat populations. This file contains data on the microsatellites analyzed i.e. total number of alleles (NA); expected heterozygosity (He); observed heterozygosity (Ho); Wright’s F- statistics and number of breeds showing deviations from Hardy–Weinberg equilibrium (HWEd) (P < 0.001). [file 12711_2015_167_MOESM2_ESM.pdf]

Additional file 2 Table S2. Descriptive statistics of genetic diversity for the 20 microsatellite markers genotyped in 29 goat populations.

Locus	NA	Freq Null Alleles	H_e	H_o	F_{IS}	F_{ST}	F_{IT}	HWEd
BM1329	12	0.024	0.763	0.755	0.020 ± 0.020	0.070 ± 0.017	0.088 ± 0.018	0
BM6506	12	0.043	0.687	0.629	0.072 ± 0.023	0.105 ± 0.023	0.169 ± 0.028	1
BM6526	21	0.018	0.737	0.721	0.015 ± 0.019	0.102 ± 0.021	0.115 ± 0.023	0
BM8125	9	0.015	0.670	0.685	-0.007 ± 0.020	0.078 ± 0.027	0.072 ± 0.035	1
CSRM60	12	0.034	0.753	0.702	0.059 ± 0.020	0.059 ± 0.013	0.114 ± 0.022	1
CSRD247	13	0.021	0.770	0.735	0.053 ± 0.017	0.093 ± 0.016	0.141 ± 0.016	1
ETH010	7	0.031	0.599	0.561	0.066 ± 0.025	0.062 ± 0.021	0.123 ± 0.031	0
ETH225	6	0.027	0.199	0.195	0.015 ± 0.030	0.023 ± 0.010	0.038 ± 0.028	0
HAUT27	12	0.035	0.736	0.661	0.076 ± 0.023	0.048 ± 0.011	0.120 ±0.023	1
ILSTS011	12	0.031	0.629	0.590	0.057 ± 0.024	0.090 ± 0.022	0.142 ± 0.030	1
INRA063	8	0.066	0.588	0.48	0.159 ± 0.032	0.068 ± 0.015	0.216 ± 0.030	1
MAF065	14	0.028	0.802	0.762	0.042 ± 0.020	0.067 ± 0.014	0.107 ± 0.023	1
MAF209	3	0.053	0.293	0.282	0.041 ± 0.035	0.060 ± 0.027	0.098 ± 0.051	0
McM527	13	0.048	0.688	0.616	0.091 ± 0.024	0.062 ± 0.016	0.148 ± 0.027	1
MM12	20	0.032	0.856	0.803	0.060 ± 0.025	0.055 ± 0.013	0.112 ± 0.025	1
OarFCB048	13	0.031	0.809	0.767	0.060 ± 0.019	0.051 ± 0.010	0.108 ± 0.018	0
OarFCB304	22	0.054	0.703	0.602	0.147 ± 0.026	0.078 ± 0.016	0.213 ± 0.030	2
SPS115	4	0.097	0.484	0.404	0.163 ± 0.040	0.070 ± 0.023	0.222 ± 0.041	2
SRCRSP08	11	0.048	0.632	0.586	0.066 ± 0.025	0.140 ± 0.032	0.196 ± 0.032	0
TGLA122	12	0.029	0.646	0.616	0.056 ± 0.025	0.070 ± 0.013	0.122 ± 0.024	0
Mean value	*11.8*	*0.038*	*0.652*	*0.608*	*0.064 ± 0.010*	*0.073 ± 0.005*	*0.133 ± 0.010*	*0.7*

NA. total number of alleles; H_e. expected heterozygosity; H_o. observed heterozygosity; F_{IS}. F_{ST} and F_{IS} Wright's F- statistics; HWd. number of breeds showing deviations from Hardy-Weinberg equilibrium (HWEd) (P < 0.001)
