# Supplementary material for: The Southwestern fringe of Europe as an important reservoir of caprine biodiversity
Source: Genet Sel Evol. 2015 Nov 5;47:86. doi: 10.1186/s12711-015-0167-8 (PMC4635977; doi:10.1186/s12711-015-0167-8)

Additional file 3 Figure S1. Correlation between FST genetic distances and geographic distances (P < 0.0001 after 10,000 permutations)

a

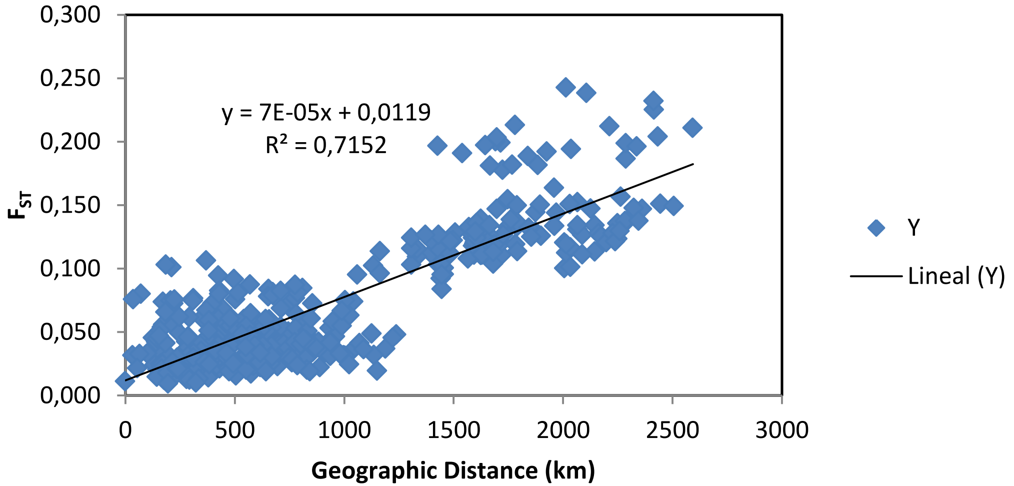

b

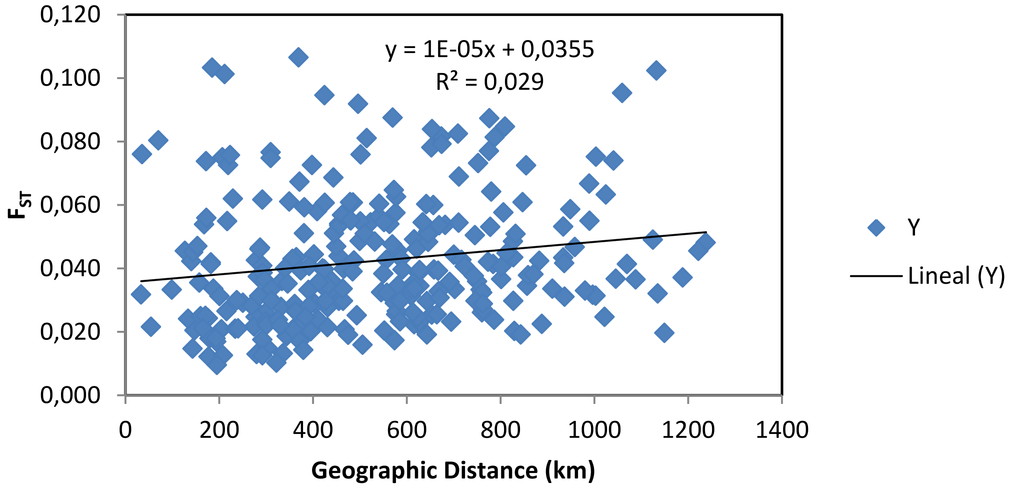

Supplement: Supplementary file 3 — 10.1186/s12711-015-0167-8 Correlation between F ST genetic distances and geographic distances (P > 0.0001 after 10,000 permutations). (a) 29 goat breeds. (b) Portuguese and Spanish breeds after removing the Canary Islands populations. [file 12711_2015_167_MOESM3_ESM.pdf]
