# Supplementary material for: The Southwestern fringe of Europe as an important reservoir of caprine biodiversity
Source: Genet Sel Evol. 2015 Nov 5;47:86. doi: 10.1186/s12711-015-0167-8 (PMC4635977; doi:10.1186/s12711-015-0167-8)

Additional file 4 Figure S2. Estimated posterior probabilities of the data for different values of K in the analysis with Structure.

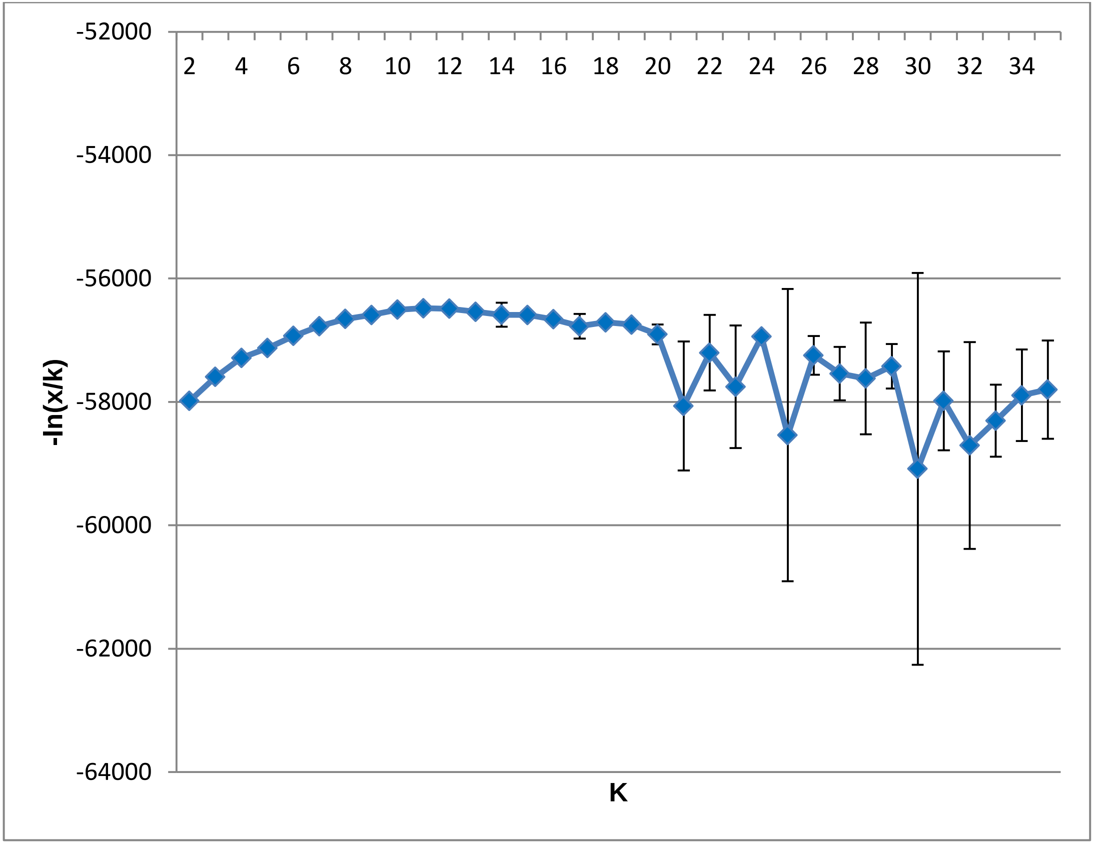

Supplement: Supplementary file 4 — 10.1186/s12711-015-0167-8 Estimated posterior probabilities of the data for different values of K in the analysis with STRUCTURE. This file contains the plots of the average likelihood of data (ln Pr(X|K)) (mean ± standard deviation of five independent runs) obtained with STRUCTURE for 29 goat breeds from Portugal and Spain for two to 35 inferred genetic clusters (K). [file 12711_2015_167_MOESM4_ESM.pdf]
