# Supplementary material for: The Southwestern fringe of Europe as an important reservoir of caprine biodiversity
Source: Genet Sel Evol. 2015 Nov 5;47:86. doi: 10.1186/s12711-015-0167-8 (PMC4635977; doi:10.1186/s12711-015-0167-8)
Supplement: Supplementary file 5 — 10.1186/s12711-015-0167-8 Proportional contribution of the clusters inferred with STRUCTURE (K = 12) to the gene pools, ancestry diversity and expected heterozygosities of 29 Portuguese and Spanish goat breeds. [file 12711_2015_167_MOESM5_ESM.pdf]

Additional file 5 Table S3. **Proportional contribution of the clusters inferred with Structure (K=12) to the gene pools, ancestry diversity and expected heterozygosities of 29 Portuguese and Spanish goat breeds.**

	Clusters													
Breed	1	2	3	4	5	6	7	8	9	10	11	12	Ancestry diversity[1]	H_e
Pirenaica	**0.172**	0.036	0.043	0.035	0.022	**0.477**	0.031	0.018	0.058	0.038	0.035	0.035	0.73	0.70
Moncaína	0.016	0.026	0.044	0.022	0.017	**0.505**	0.056	0.034	0.066	0.042	0.074	0.098	0.72	0.69
Azpi Gorri	0.030	0.053	0.104	0.018	0.016	0.012	**0.618**	0.011	0.017	0.019	0.039	0.064	0.60	0.66
Blanca de Rasquera	0.023	0.055	0.045	0.016	0.010	0.022	0.022	0.013	**0.716**	0.021	0.020	0.036	0.48	0.63
Guadarrama	0.039	0.120	0.120	0.050	0.011	0.011	0.074	0.010	0.109	0.071	0.130	**0.255**	0.86	0.61
Retinta	0.018	0.086	**0.173**	0.073	0.032	0.033	**0.190**	0.017	0.022	0.129	0.094	0.132	0.88	0.69
Verata	0.018	0.132	0.119	0.016	0.013	0.036	0.083	0.011	0.068	**0.153**	0.063	**0.289**	0.84	0.65
Blanca Andaluza	0.019	0.128	0.135	0.092	0.040	0.028	0.100	0.035	0.046	0.072	**0.217**	0.089	0.88	0.67
Celtibérica	0.025	0.095	**0.158**	0.065	0.030	0.021	0.054	0.021	0.062	0.061	**0.181**	**0.226**	0.87	0.66
Blanca Celtibérica	**0.610**	0.023	0.012	0.032	0.059	0.025	0.026	0.008	0.056	0.052	0.063	0.033	0.61	0.65
Malagueña	0.029	0.040	**0.186**	0.086	0.034	0.017	0.060	0.021	0.039	0.027	0.115	**0.346**	0.81	0.68
Murciano-Granadina	0.016	0.024	0.046	0.030	0.011	0.010	0.039	0.013	0.037	0.032	**0.677**	0.064	0.53	0.66
Florida	0.026	0.131	**0.213**	0.076	0.024	0.030	0.046	0.010	0.028	0.076	0.079	**0.261**	0.85	0.70
Payoya	0.017	0.082	0.040	0.018	0.021	0.013	0.017	0.014	0.018	**0.616**	0.020	0.125	0.59	0.67
Negra Serrana	0.012	0.041	**0.319**	0.036	0.012	0.009	0.026	0.019	0.019	0.020	0.057	**0.430**	0.70	0.66
Formentera	0.025	0.016	0.025	0.027	0.026	0.032	0.068	0.011	0.020	**0.680**	0.053	0.016	0.53	0.60
Pitiusa	0.022	0.081	0.073	0.070	**0.407**	0.011	0.059	0.014	0.020	0.059	0.135	0.050	0.79	0.65
Mallorquina	0.008	0.033	0.033	**0.574**	**0.158**	0.008	0.015	0.008	0.061	0.015	0.036	0.051	0.64	0.65
Ajuí	0.010	0.013	0.014	0.011	0.012	0.010	0.016	**0.865**	0.018	0.008	0.010	0.014	0.25	0.65
Majorera	0.018	0.011	0.025	0.011	0.009	0.011	0.010	**0.852**	0.010	0.013	0.010	0.020	0.27	0.64
Palmera	0.003	0.004	0.004	0.004	0.003	0.003	0.004	**0.959**	0.004	0.004	0.004	0.005	0.08	0.50
Tenerife Norte	0.007	0.008	0.014	0.009	0.007	0.007	0.010	**0.892**	0.009	0.008	0.017	0.013	0.20	0.60
Tenerife Sur	0.008	0.010	0.009	0.008	0.006	0.007	0.018	**0.907**	0.006	0.007	0.008	0.006	0.18	0.60
Bravia	0.009	**0.751**	0.046	0.020	0.010	0.008	0.049	0.009	0.022	0.018	0.022	0.034	0.43	0.63
Serpentina	0.023	**0.183**	**0.150**	0.046	0.025	0.067	0.089	0.022	0.047	0.095	0.100	**0.153**	0.88	0.67
Algarvia	0.014	0.091	**0.545**	0.046	0.039	0.016	0.031	0.018	0.026	0.064	0.060	0.050	0.68	0.68
Charnequeira	0.043	**0.179**	**0.118**	0.091	0.028	0.011	0.044	0.030	0.094	0.051	**0.125**	**0.186**	0.88	0.69
Serrana	0.028	0.099	**0.172**	0.090	0.025	0.078	0.076	0.010	0.051	**0.142**	0.080	**0.149**	0.89	0.67
Preta de Montesinho	0.040	0.073	**0.284**	0.052	0.030	0.020	0.099	0.021	0.088	0.103	0.080	0.109	0.86	0.67

[1]Ancestry diversity for each breed was calculated as $1-\Sigma(qk)^2$, where qk is an average fraction of the genetic ancestry of the breed belonging to genetic clusters k, identified in STRUCTURE analysis.
