# Supplementary material for: The Southwestern fringe of Europe as an important reservoir of caprine biodiversity
Source: Genet Sel Evol. 2015 Nov 5;47:86. doi: 10.1186/s12711-015-0167-8 (PMC4635977; doi:10.1186/s12711-015-0167-8)
Supplement: Supplementary file 6 — 10.1186/s12711-015-0167-8 Proportional contribution of the clusters inferred with STRUCTURE (K = 24) to the gene pool of the 29 Portuguese and Spanish goat populations. Contributions of the most important clusters per population are represented in bold. Genetic clusters with a contribution of less than 10 % in any of the 29 goat populations are shown in grey. [file 12711_2015_167_MOESM6_ESM.pdf]

Additional file 6 Table S4. Proportional contribution of the clusters inferred with Structure (K=24) to the gene pools of 29 Spanish and Portuguese goat populations.

	Clusters																							
Breed	1	2	3	4	5	6	7	8	9	10	11	12	13	14	15	16	17	18	19	20	21	22	23	24
Pirenaica	0.006	0.006	**0.461**	0.006	0.033	0.006	0.028	0.006	0.029	0.142	0.037	0.016	0.006	0.036	0.006	0.006	0.013	0.060	0.036	0.038	0.006	0.006	0.006	0.006
Moncaína	0.005	0.004	**0.465**	0.004	0.078	0.004	0.019	0.004	0.055	0.012	0.050	0.012	0.004	0.040	0.004	0.004	0.030	0.064	0.023	0.099	0.004	0.004	0.004	0.004
Azpi Gorri	0.003	0.003	0.009	0.003	0.038	0.003	0.013	0.003	**0.611**	0.021	0.113	0.011	0.003	0.017	0.003	0.003	0.009	0.014	0.051	0.058	0.003	0.003	0.003	0.003
Blanca de Rasquera	0.002	0.002	0.017	0.002	0.018	0.002	0.012	0.002	0.020	0.018	0.049	0.007	0.002	0.018	0.002	0.002	0.011	**0.716**	0.053	0.034	0.002	0.002	0.002	0.002
Guadarrama	0.004	0.004	0.008	0.004	0.124	0.004	0.039	0.004	0.067	0.027	**0.165**	0.008	0.004	0.065	0.004	0.004	0.007	0.095	0.115	**0.232**	0.004	0.004	0.004	0.004
Retinta	0.004	0.004	0.022	0.004	0.091	0.004	0.066	0.004	**0.174**	0.013	**0.193**	0.024	0.004	0.123	0.004	0.004	0.014	0.019	0.084	0.128	0.004	0.004	0.004	0.004
Verata	0.005	0.004	0.032	0.004	0.063	0.004	0.011	0.004	0.074	0.013	0.124	0.010	0.004	0.148	0.004	0.004	0.008	0.063	0.120	**0.283**	0.004	0.004	0.004	0.004
Blanca Andaluza	0.005	0.005	0.019	0.005	**0.223**	0.005	0.077	0.005	0.092	0.013	**0.163**	0.025	0.005	0.063	0.005	0.005	0.029	0.041	0.110	0.091	0.005	0.005	0.005	0.005
Celtibérica	0.004	0.004	0.015	0.004	0.181	0.004	0.049	0.004	0.048	0.018	**0.177**	0.023	0.004	0.055	0.004	0.004	0.017	0.056	0.084	**0.225**	0.004	0.004	0.004	0.004
Blanca Celtibérica	0.004	0.003	0.023	0.003	0.063	0.003	0.030	0.003	0.026	**0.571**	0.012	0.054	0.003	0.056	0.003	0.003	0.007	0.056	0.024	0.037	0.003	0.003	0.003	0.003
Malagueña	0.004	0.004	0.012	0.004	0.110	0.004	0.063	0.004	0.054	0.019	**0.211**	0.026	0.004	0.024	0.004	0.004	0.016	0.032	0.036	**0.349**	0.004	0.004	0.004	0.004
Murciano-Granadina	0.005	0.004	0.008	0.004	**0.650**	0.004	0.022	0.004	0.031	0.011	0.053	0.008	0.004	0.030	0.004	0.004	0.010	0.034	0.022	0.073	0.004	0.004	0.004	0.004
Florida	0.005	0.005	0.022	0.005	0.078	0.005	0.062	0.005	0.043	0.017	**0.208**	0.018	0.005	0.071	0.005	0.005	0.008	0.024	0.121	**0.270**	0.005	0.005	0.005	0.005
Payoya	0.003	0.003	0.010	0.003	0.019	0.003	0.014	0.003	0.016	0.012	0.040	0.015	0.003	**0.606**	0.003	0.003	0.011	0.014	0.077	0.127	0.003	0.003	0.003	0.003
Negra Serrana	0.004	0.004	0.007	0.004	0.053	0.004	0.026	0.004	0.025	0.008	**0.294**	0.008	0.004	0.020	0.004	0.004	0.016	0.016	0.039	**0.443**	0.004	0.004	0.004	0.004
Formentera	0.003	0.003	0.023	0.003	0.055	0.003	0.019	0.003	0.070	0.018	0.032	0.017	0.003	**0.680**	0.003	0.003	0.009	0.016	0.013	0.013	0.003	0.003	0.003	0.003
Pitiusa	0.003	0.003	0.008	0.003	0.142	0.003	0.073	0.003	0.056	0.015	0.125	**0.340**	0.003	0.060	0.003	0.003	0.012	0.017	0.071	0.049	0.003	0.003	0.003	0.003
Mallorquina	0.002	0.002	0.007	0.002	0.043	0.002	**0.513**	0.002	0.014	0.006	0.064	0.150	0.002	0.014	0.002	0.002	0.007	0.061	0.028	0.066	0.002	0.002	0.002	0.002
Ajuí	0.003	0.003	0.008	0.003	0.008	0.003	0.008	0.003	0.013	0.007	0.011	0.008	0.003	0.006	0.003	0.003	**0.856**	0.014	0.010	0.011	0.003	0.003	0.003	0.003
Majorera	0.004	0.004	0.007	0.004	0.008	0.004	0.009	0.004	0.008	0.013	0.019	0.007	0.004	0.010	0.004	0.004	**0.837**	0.008	0.009	0.016	0.004	0.004	0.004	0.004
Palmera	0.002	0.002	0.003	0.002	0.003	0.002	0.003	0.002	0.003	0.002	0.003	0.002	0.002	0.003	0.002	0.002	**0.949**	0.003	0.003	0.004	0.002	0.002	0.002	0.002
Tenerife Norte	0.002	0.002	0.005	0.002	0.014	0.002	0.006	0.002	0.008	0.005	0.012	0.005	0.002	0.006	0.002	0.002	**0.888**	0.007	0.007	0.011	0.002	0.002	0.002	0.002
Tenerife Sur	0.002	0.002	0.005	0.002	0.006	0.002	0.006	0.002	0.016	0.006	0.007	0.004	0.002	0.005	0.002	0.002	**0.899**	0.005	0.007	0.005	0.002	0.002	0.002	0.002
Bravia	0.002	0.002	0.006	0.002	0.022	0.002	0.014	0.002	0.048	0.006	0.063	0.007	0.002	0.015	0.002	0.002	0.007	0.019	**0.734**	0.034	0.002	0.002	0.002	0.002
Serpentina	0.003	0.003	0.052	0.003	0.100	0.003	0.033	0.003	0.083	0.016	**0.185**	0.016	0.003	0.094	0.003	0.003	0.017	0.049	**0.166**	**0.153**	0.003	0.003	0.003	0.003
Algarvia	0.004	0.004	0.011	0.004	0.056	0.004	0.029	0.004	0.027	0.011	**0.560**	0.031	0.004	0.056	0.004	0.004	0.015	0.022	0.083	0.049	0.004	0.004	0.004	0.004
Charnequeira	0.004	0.004	0.008	0.004	0.131	0.004	0.068	0.004	0.038	0.034	0.136	0.019	0.004	0.047	0.004	0.004	0.026	0.087	**0.181**	**0.181**	0.004	0.004	0.004	0.004
Serrana	0.004	0.004	0.065	0.004	0.079	0.004	0.073	0.004	0.074	0.020	**0.192**	0.016	0.004	0.141	0.004	0.004	0.008	0.046	0.085	0.156	0.004	0.004	0.004	0.004
Preta de Montesinho	0.003	0.003	0.014	0.003	0.087	0.003	0.036	0.003	0.093	0.033	**0.319**	0.020	0.003	0.094	0.003	0.003	0.017	0.079	0.062	0.108	0.003	0.003	0.003	0.003

Contributions of the most important clusters per population are represented in bold. In gray: genetic clusters with a contribution of less than 10% in any of the 29 goat populations.
