# Supplementary material for: The Southwestern fringe of Europe as an important reservoir of caprine biodiversity
Source: Genet Sel Evol. 2015 Nov 5;47:86. doi: 10.1186/s12711-015-0167-8 (PMC4635977; doi:10.1186/s12711-015-0167-8)

Additional file 7 Figure S3. UPGMA clustering of the ultra-metric distance matrix obtained from the conversion of the average genotype membership coefficients (Q) in each cluster (K = 3) into genetic distances.

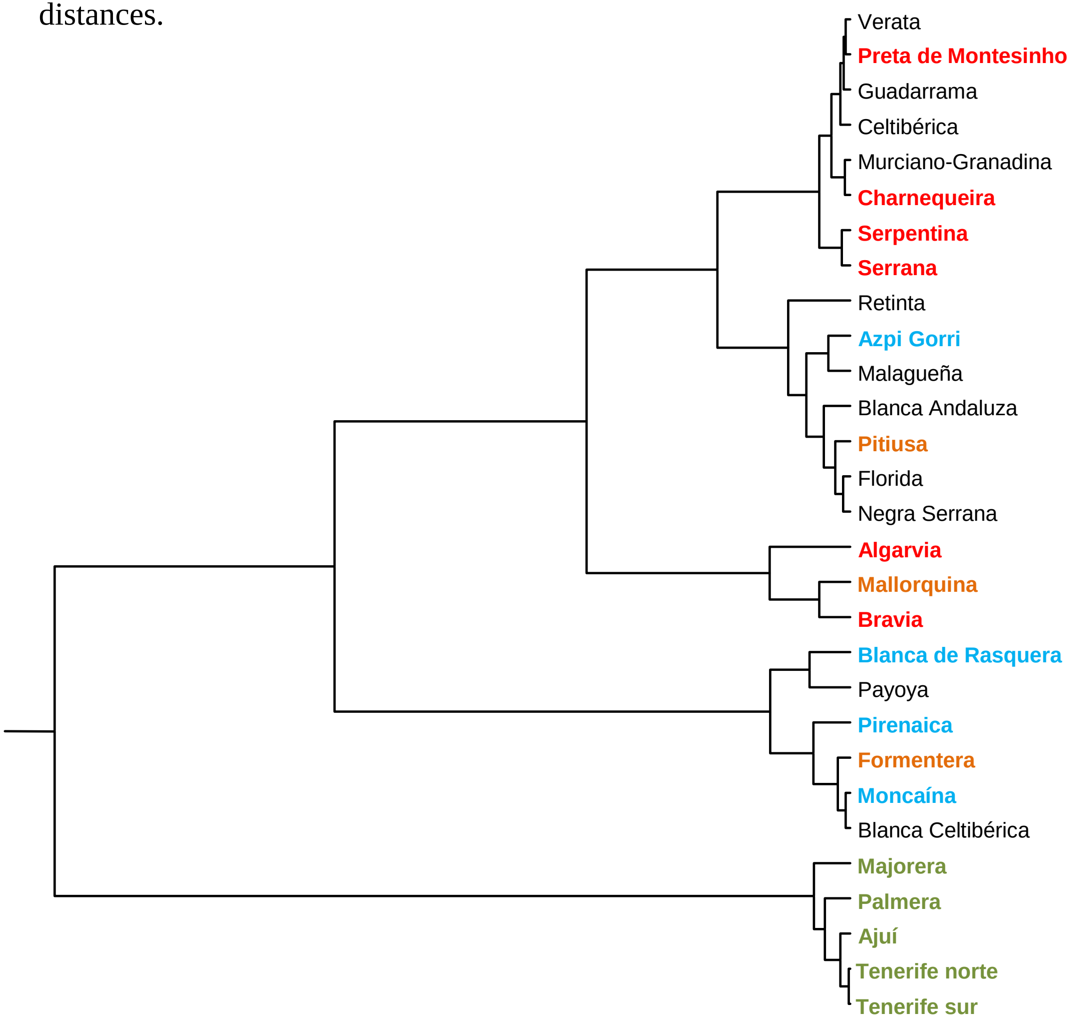

Supplement: Supplementary file 7 — 10.1186/s12711-015-0167-8 UPGMA clustering of the ultra-metric distance matrix obtained from the conversion of the average genotype membership coefficients (Q) in each cluster (K = 3) into genetic distances. [file 12711_2015_167_MOESM7_ESM.pdf]
