# Supplementary material for: The Southwestern fringe of Europe as an important reservoir of caprine biodiversity
Source: Genet Sel Evol. 2015 Nov 5;47:86. doi: 10.1186/s12711-015-0167-8 (PMC4635977; doi:10.1186/s12711-015-0167-8)
Supplement: Supplementary file 8 — 10.1186/s12711-015-0167-8 Demographic information on the 29 Portuguese and Spanish goat populations. This file includes the name of the populations, acronyms used, existence of Herdbook, year of the establishment of the Herdbook, census, geographic distribution (GD), use of artificial insemination (AI), existence of gene bank (GB), existence of conservation programs (CP) and existence of selection programs (SP). Source: http://www.magrama.gob.es/es/ganaderia/temas/zootecnia/razas-ganaderas/razas/catalogo/ (Accessed on 15 Aug 2015). [file 12711_2015_167_MOESM8_ESM.pdf]

Additional file 8. Demographic information on 29 Portuguese and Spanish goat populations. Name of the populations, acronyms, existence of Herdbok, year of Herdbook's establishment, census, geographic distribution (GD), use of artificial insemination (AI), existence of gene bank (GB), existence of conservation programs (CP), existence of selection programs (SP).

Source: http://www.magrama.gob.es/es/ganaderia/temas/zootecnia/razas-ganaderas/razas/catalogo/ (Accessed: 15/08/2015)

Population	Acronym	Herdbook	Year	Census	Risk Status	GD[5]	AI	GB	CP	SP
**SPAIN**										
**Pirenaica**	PIR	YES	2000	1,627[3]	Endangered	ARA, CAT, CL, N,	NO	NO	YES	YES
**Moncaína**	MON	YES	2000	2,693[3]	Endangered	ARA	NO	NO	YES	YES
**Azpi Gorri**	AZ	YES	2000	1,607[3]	Endangered	PV, N	NO	NO	YES	YES
**Blanca de Rasquera**	RAS	NO	2010	5,000[3]	Endangered	CAT	NO	NO	YES	YES
Guadarrama	GUAD	YES	1999	9,212[3]	Endangered	M, CL	NO	NO	YES	YES
Retinta	RET	YES	2003	2,307[3]	Endangered	EX	NO	NO	YES	YES
Verata	VERA	YES	2000	8,738[3]	Endangered	CL, CV, EX	NO	NO	YES	YES
Blanca Andaluza	BLANCA	YES	2004	8,642[3]	Endangered	A, EX, CV	NO	NO	YES	YES
Celtibérica	CELTIB	YES*	1995	7,904[3]	Endangered	A, ARA, CLM, MUR, CV	NO	YES	YES	YES
Blanca Celtibérica	BC			<100						
Malagueña	MALAG	YES	1983	40,872[3]	Not at risk	IP	YES	YES	NO	YES
Murciano-Granadina	MG	YES	1975	99,335[3]	Not at risk	IP	YES	YES	NO	YES
Florida	FLO	YES	1996	24,2493	Not at risk	A, EX, CLM, CL	YES	YES	NO	YES
Payoya	PAY	YES	1995	6,905[3]	Endangered	A, CAT	YES	YES	YES	YES
Negra Serrana	SER	YES	2003	4,715[3]	Endangered	A, CLM	NO	NO	YES	YES
**Formentera[1]**	FOR	YES*	2008	225[3,*]	Endangered	BI	YES	YES	YES	YES
**Pitiusa[1]**	IB									
**Mallorquina[1]**	MALL	YES	1996	236[3]	Endangered	BI	NO	YES	YES	YES
**Ajuí[2]**	AJ	NO	-	1,700[3]	Endangered	CI	NO	NO	NO	NO
**Majorera[2]**	MFV	YES	2011	12,832[3]	Not at risk	CI	NO	NO	NO	YES
**Palmera[2]**	PAL	YES	2007	9,158[3]	Not at risk	CI	NO	NO	NO	YES
**Tenerife Norte[2]**	TFN	YES*	2007	4,705[3,*]	Not at risk	CI	NO	NO	YES	YES
**Tenerife Sur[2]**	TFS									
**PORTUGAL**										
**Bravia**	BR	YES	1996	9,768[4]	Endangered	N	NO	YES	YES	YES
**Serpentina**	SP	YES	1991	4,816[4]	Endangered	S	NO	YES	YES	YES
**Algarvia**	AL	YES	1986	3,991[4]	Endangered	S	NO	YES	YES	YES
**Charnequeira**	CH	YES	1986	4,403[4]	Endangered	C, S	NO	YES	YES	YES
**Serrana**	SR	YES	1986	18,607[4]	Not at risk	N, C	YES	YES	YES	YES
**Preta de Montesinho**	PM	YES	2009	707[4]	Endangered	N	NO	YES	YES	YES

[1]Breeds from the Balearic Islands; [2]Breeds from the Canary Islands; [3]https://aplicaciones.magrama.es/arca-webapp/flujos.html?_flowId=catalogoRazas-flow&_flowExecutionKey=e1s (Census at 12/31/2014 considering registered animals); [*]Both populations are registered in the same herdbook.

[4]http://www.dgv.min-agricultura.pt/xeov21/attachfileu.jsp?look_parentBoui=3820310&att_display=n&att_download=y (Census at 12/31/2014 considering registered animals)

[5]SPAIN: A: Andalucía, ARA: Aragón, BI: Balearic Islands, CAT: Cataluña, CI: Canary Islands, CL: Castilla y León, CLM: Castilla-La Mancha, CV: Comunidad Valenciana, EX: Extremadura, IP: Iberian Peninsula, M: Madrid, MUR: Murcia, N: Navarra, PV: País Vasco. PORTUGAL: N: North; C: Center; S: South.
